# In vivo dynamic motion characteristics of the lower lumbar spine: L4–5 lumbar degenerative disc diseases undergoing unilateral or bilateral pedicle screw fixation combined with TLIF

**DOI:** 10.1186/s13018-019-1198-6

**Published:** 2019-06-07

**Authors:** Tao Nie, De-jian Chen, Benyu Tang, Quanwei Song, Xuqiang Liu, Bin Zhang, Min Dai, Guoan Li, Zongmiao Wan

**Affiliations:** 1 Department of Orthopedics, The First Affiliated Hospital of Nanchang University, 17 Yongwai Street, Nanchang, 330006 Jiangxi People’s Republic of China; 2 Bioengineering Lab, Department of Orthopaedic Surgery, Massachusetts General Hospital/Harvard Medical School, 55 Fruit St., GRJ 1215, Boston, MA 02114 USA

**Keywords:** Unilateral pedicle screw fixation, Bilateral pedicle screw fixation, Lumbar spine, In vivo ROM, Adjacent segment

## Abstract

**Objective:**

To evaluate the short-term in vivo dynamic motion characteristics of the lower lumbar spine (L3–S1) after unilateral pedicle screw fixation (UPSF) or bilateral pedicle screw fixation (BPSF) combined with TLIF for treatment of L4–5 lumbar degenerative disc diseases (DDD).

**Methods:**

Twenty-eight patients were recruited (13 UPSF, 15 BPSF). Each patient was CT-scanned to construct 3D models of the L3–S1 vertebrae. The dual fluoroscopic imaging system (DFIS) was then used to image the lumbar spine while the patient performed seven functional activities (upright standing, maximum extension, flexion, left–right twist, and left–right bend). The in vivo vertebral positions were reproduced using the 3D vertebral models and DFIS images. The ranges of motion (ROMs) of L3–4, L4–5, and L5–S1 segments were analyzed.

**Results:**

At the index L4–5 segment, the primary ROM of left–right twist of the UPSF group (2.11 ± 0.52°) was significantly larger (*p* = 0.000) than the BPSF group (0.73 ± 0.32°). At the proximal adjacent L3–4 segment, the primary ROMs of left–right twist, and left–right bend of the UPSF group (2.16 ± 0.73°, 2.28 ± 1.03°) were significantly less (*p* = 0.003, 0.023) than the BPSF group (3.17 ± 0.88, 3.12 ± 1.04°), respectively. However, at distal adjacent L5–S1 segment, no significant difference was found between the two groups during all activities.

**Conclusions:**

The ROM in left–right twisting of UPSF group was significantly larger compared with BPSF group at the index level in the short term. The UPSF has less impact on the cranial adjacent level (L3–4) in left–right twisting and bending activities compared to the BPSF. The data implied that the UPSE and BPSF combined with TLIF would result in different biomechanics in the index and cranial adjacent segment biomechanics. Long-term follow-up studies are necessary to compare the clinical outcomes of the two surgeries.

**Electronic supplementary material:**

The online version of this article (10.1186/s13018-019-1198-6) contains supplementary material, which is available to authorized users.

## Introduction

Lumbar degenerative disc diseases (DDD) have become common diseases of orthopedics, and epidemiological studies have revealed that lumbar DDD is often found in the lower lumbar levels of L4–5 and L5–S1 [1, 2]. Bilateral pedicle screw fixation (BPSF) combined with transforaminal lumbar interbody fusion (TLIF) is a widely used method to treat lumbar DDD which has a variety of advantages, such as excellent stability, great fixation intensity, and high fusion rate [3, 4]. However, some studies have shown that excessive fixation intensity of BPSF can cause more clinically adverse effects, such as adjacent segment degeneration [5, 6]. To address these deficiencies, unilateral pedicle screw fixation (UPSF) combined with TLIF has become a focused issue in treating with a single segment of lumbar DDD recently [7]. At present, biomechanical studies on unilateral and bilateral pedicle screw fixations are still controversial. Most in vitro studies have measured lumbar segment motion by applying flexion–extension, bending, and twisting torques, with or without a compressive load. Slucky et al. [8] reported the lumbar segment flexibility and motion range of UPSF combined with TLIF group were significantly better than that of the BPSF group. However, Goel et al. [9] have shown that the stability of UPSF was not good as the BPSF group thought it allows a greater range of segmental lumbar motion. Accurate in vivo dynamic motion characteristics of L3–S1 in 6 degrees of freedom (6DOF) after UPSF or BPSF with TLIF are still not clearly described in the literature. Recently, a 3D fluoroscopic imaging technique has been applied to investigate 6DOF lumbar kinematics during various dynamic axial rotation which has the accuracy of 0.3 mm in translations and 0.7°in rotations [10–13].

In this study, we combined the dual fluorescence imaging system (DFIS) with 2 dimensions to 3 dimensions (2D-3D) matching techniques to study the short-term in vivo dynamic motion characteristics of low lumbar spine after unilateral and bilateral pedicle screw fixation combined with TLIF for L4–5 lumber DDD. We hypothesized that the UPSF is not as rigid as the BPSF in constraining the index segment, it could result in less effect on the adjacent levels compared to the BPSF technique.

## Materials and methods

### Patient characteristics

Thirty-one patients with lumbar DDD who suffered from unilateral radiculopathy and were scheduled to undergo unilateral or bilateral pedicle screw fixation with TLIF only at L4–5 lumbar spine were recruited between July 2016 and May 2018. The inclusion criteria were (1) L4–5 lumbar DDD, (2) suffered from unilateral radicular symptoms in a single lower limb and underwent unilateral TLIF combined with unilateral or bilateral pedicle screw fixation, (3) not necessary to perform spinal decompression by windowing or facet joint resection at contralateral crypt, and (4) 1–3 months after surgery. The exclusion criteria were (1) lumber spondylolisthesis grade > I (according to Meyerding classification), (2) lumbar spondylolysis, (3) presence of postoperative infection, (4) osteoporosis patient, (5) other lumbar surgeries before. The UPSF and BPSF were used according to surgeon’s and patients’ choice, and all patients underwent unilateral facetectomy and hemilaminectomy on the symptomatic side.

Of the 31 recruited patients, three patients were excluded from this study: two lost follow-up visit postoperatively, and one was checked out with postoperative infection at surgical segment at 1 month postoperative. Finally, 28 patients were included in this study, where 13 underwent unilateral (6 left and 7 right) and 15 bilateral pedicle screw fixation combined with unilateral TLIF at L4–5 (mean age 51 years, range from 29 to 65 years old). The average testing time was 6 weeks postoperatively, and there were no postoperative complications at the postoperative follow-up (Table 1).

**Table 1:**
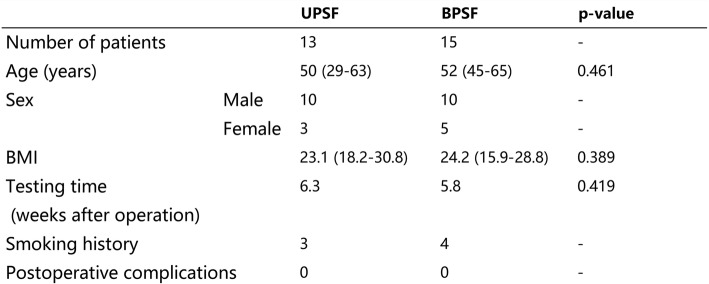
Comparison of the demographic data of the patients in the UPSF and the BPSF groups

All of the unilateral TLIF used single polyetheretherketone (PEEK) cage (Medtronic, USA) stabilized with two or four pedicle screws (China Kanghui Holdings or Huasheng Medical Equipment CO., LTD).

### Three-dimensional models of L3 to S1

Lumbar segments of each subject were scanned using a computed tomography (CT) (Light-Speed Pro16, General Electric, Waukesha, WI, USA) in a relaxed, supine position with high-resolution axial plane images. Images were obtained with a thickness of 0.75 mm, without a gap, and with a resolution of 512 × 512 pixels.

**Fig. 1:**
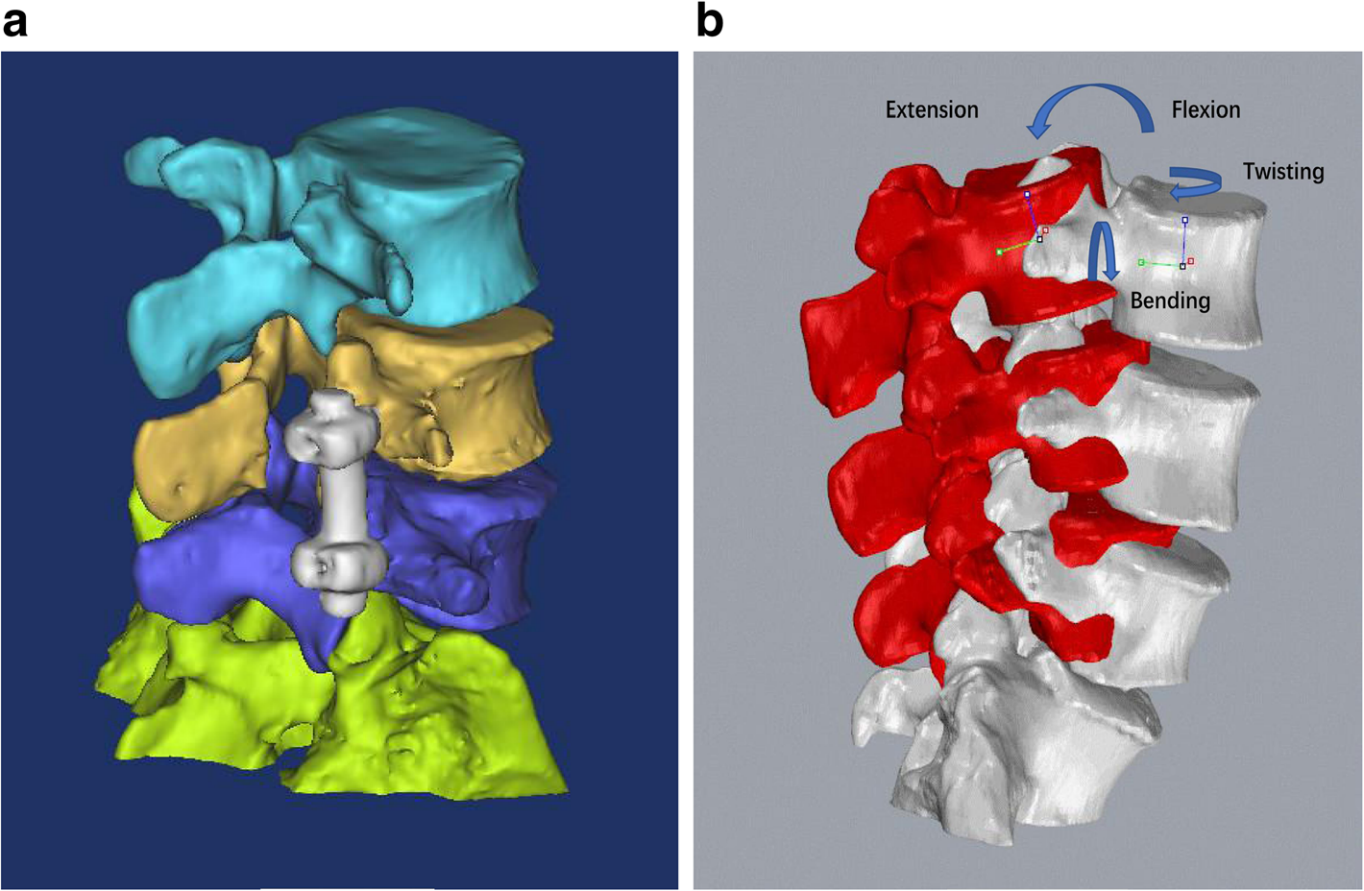
**a** Construction of 3-dimensional anatomic models of L–S1 vertebrae and unilateral pedicle screw fixation. **b** Coordinate systems were established at the volumetric center of the vertebral body to measure the relative motion between vertebrae

The CT images were imported into a solid modeling software (Mimics version 17.0, Materialise, Belgium) to construct 3-dimensional anatomic vertebral models of L3, L4, L5, partial S1, and pedicle screw fixations using an established, validated protocol [14]. Figure 1 a shows a typical 3D model of the lumbar segments including L3 to S1 and unilateral pedicle screw fixation.

### Virtual location establishment of vertebral positions

**Fig. 2:**
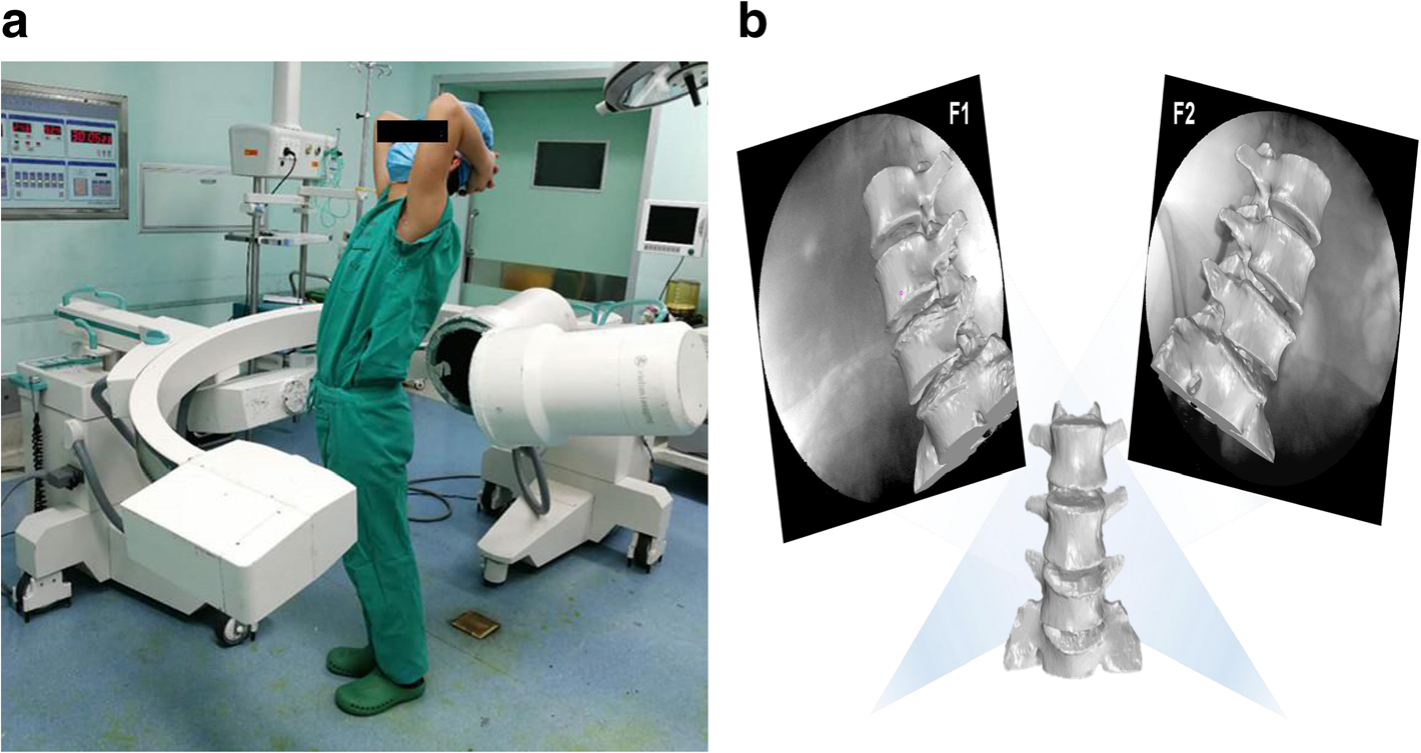
**a** Subject performing 7 functional postures of the torso (upright standing, maximum extension, flexion, left–right twist and left–right bend) in the capture of the dual fluoroscopic imaging system. **b** In the solid modeling software (Rhinoceros 5.0), the in vivo vertebral positions were reproduced

The lower lumbar spine of the patients was imaged using a dual fluoroscopic imaging system at 7 weight-bearing postures of the torso: upright standing, maximum extension, flexion, left–right twist and left–right bend (Fig. 2a). Two fluoroscopes (Ziehm 8000, Ziehm imaging, Nuremberg, Germany) were positioned with their image intensifiers perpendicular to each other in order to capture orthogonal images of the spine segments along the motion path. The interval between the x-ray source and the receiver of each fluoroscope was approximately 1 m to allow the subject to perform the movement actively when imaged by the fluoroscopes simultaneously. Each subject was exposed to approximately 30 pairs of fluoroscopic projections during the abovementioned seven motions and the entire experiment took about 10 min.

The virtual vertebral positions during the seven motions were reproduced in the solid modeling software (Rhinoceros version 5.0, Robert McNeel & Associates, Seattle, WA, USA) using the 3D models of the vertebrae and the orthogonal fluoroscopic images. Using an established protocol, the CT image-based 3D models of the vertebrae were independently translated and rotated in 6 degrees of freedom (6DOF) in increments of 0.01 mm and 0.01^o^, respectively, until their projection outlines matched the osseous contours captured on the 2 fluoroscopic images (Fig. 2b) [14–16].

### Establishment of vertebral coordinate system and measurement of the relative motions of the vertebrae

After in vivo vertebral positions were reproduced by using the 3D anatomic vertebral models, the relative motions of the vertebrae were measured using local right-hand Cartesian coordinate systems constructed at each vertebra [14] (Fig. 1b). The geometric center of the vertebra was chosen as the origin of the coordinate system. The *X*-axis was in the frontal plane and pointed to the left, the *Y*-axis was in the sagittal plane and pointed posteriorly, and the *Z*-axis was vertical to the X–Y plane and pointed proximally.

In this study, we calculated three relative vertebral levels motions (L3–4, L4–5, and L5–S1) from the proximal vertebrae relative to the distal vertebrae. Three translations were defined as the motions of the proximal vertebral coordinate system origins in the distal coordinate system: left–right, anterior–posterior, and proximal–distal translations. Three rotations were defined as the orientations of the proximal vertebral coordinate systems in the distal vertebral coordinate systems using Euler angles (in X–Y–Z sequence): flexion–extension, left–right bending, and left–right twisting rotations (Fig. 1b). For each torso posture, the range of motion (ROM) data consisted of the primary rotations and the coupled translations and rotations in all 6DOF.

### Statistical analysis

Two-way repeated measures analysis of variance (ANOVA) was used to compare the differences of ROMs during different lumbar vertebral motions (L3–4, L4–5, and L–S1) between the BPSF and the UPSF groups. When a statistically significant difference was detected, a post hoc Newman–Keuls test was performed. The statistical significance was set when *p* < 0.05. Statistical analyses were performed using SPSS 24.0 statistical software (SPSS Inc., Chicago, IL, USA).

## Results

**Fig. 3:**
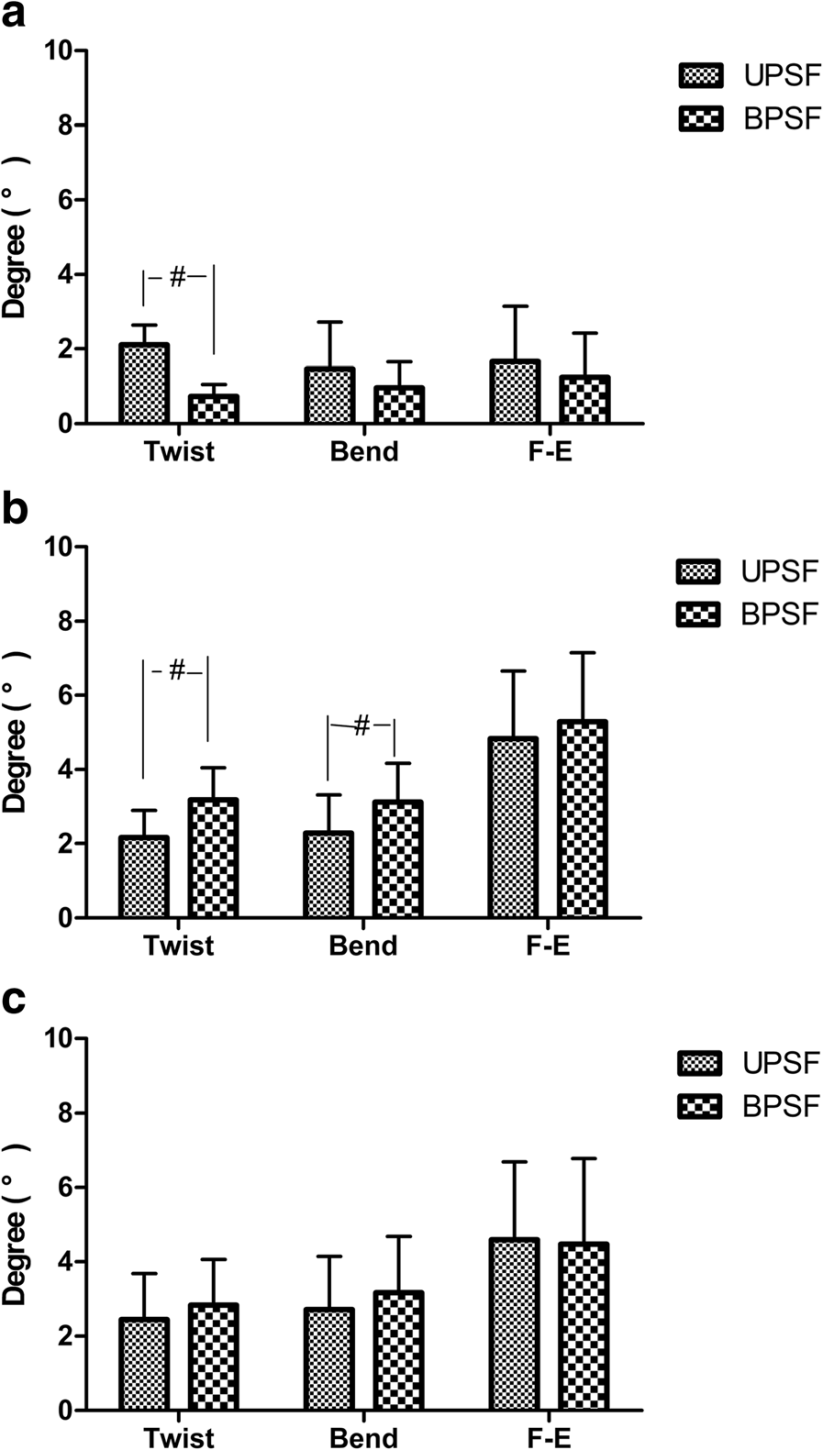
Primary ranges of motion (ROM) during bend (left–right bending), twist (left–right twisting), and flexion–extension (from maximum flexion to maximum extension) **a** at the surgical segment (L4–5), **b** at the cranial adjacent segment (L3–4), **c** at the caudal adjacent segment (L5–S1). Number sign represents significance (*p* < 0.05) for unilateral pedicle screw fixation (UPSF) at primary ROM when compared with the data of bilateral pedicle screw fixation (BPSF)

No significant differences were found between the UPSF group and BPSF group in terms of age, BMI, and testing time (Table 1). In the present study, there were 6 left fixations and 7 right fixations in the UPSF group. First, we compared the 6DOF relative ROMs at L3–4, L4–5, and L5–S1 segments, there were no significant differences between the two fixed sides (Table 2). Then, we compared the VAS, ODI, and JOA scores at preoperative, 1 month, and 3 months postoperative follow-up of the two fixed sides with no significant differences found which showed in the Additional file 1. Finally, we compare the 6DOF relative ROMs of the UPSF and BPSF groups. During left–right twisting, at the surgical segment L4–5, the ROM of primary rotation of the UPSF group was 2.11 ± 0.52° that was significantly (*p* = 0.000) larger than that (0.73 ± 0.32°) of the BPSF group. During the left–right bending, the ROM of the UPSF group (1.47 ± 1.25°) was similar to that (0.96 ± 0.69°) of the BPSF group (*p* = 0.189). During the flexion–extension, the primary ROM was also similar (*p* = 0.722) between the UPSF and BPSF groups (1.67 ± 1.48° and 1.24 ± 1.17°, respectively) (Fig. 3a). The coupled translations and rotations of the two groups had no significant differences (Table 3).

**Table 2:**
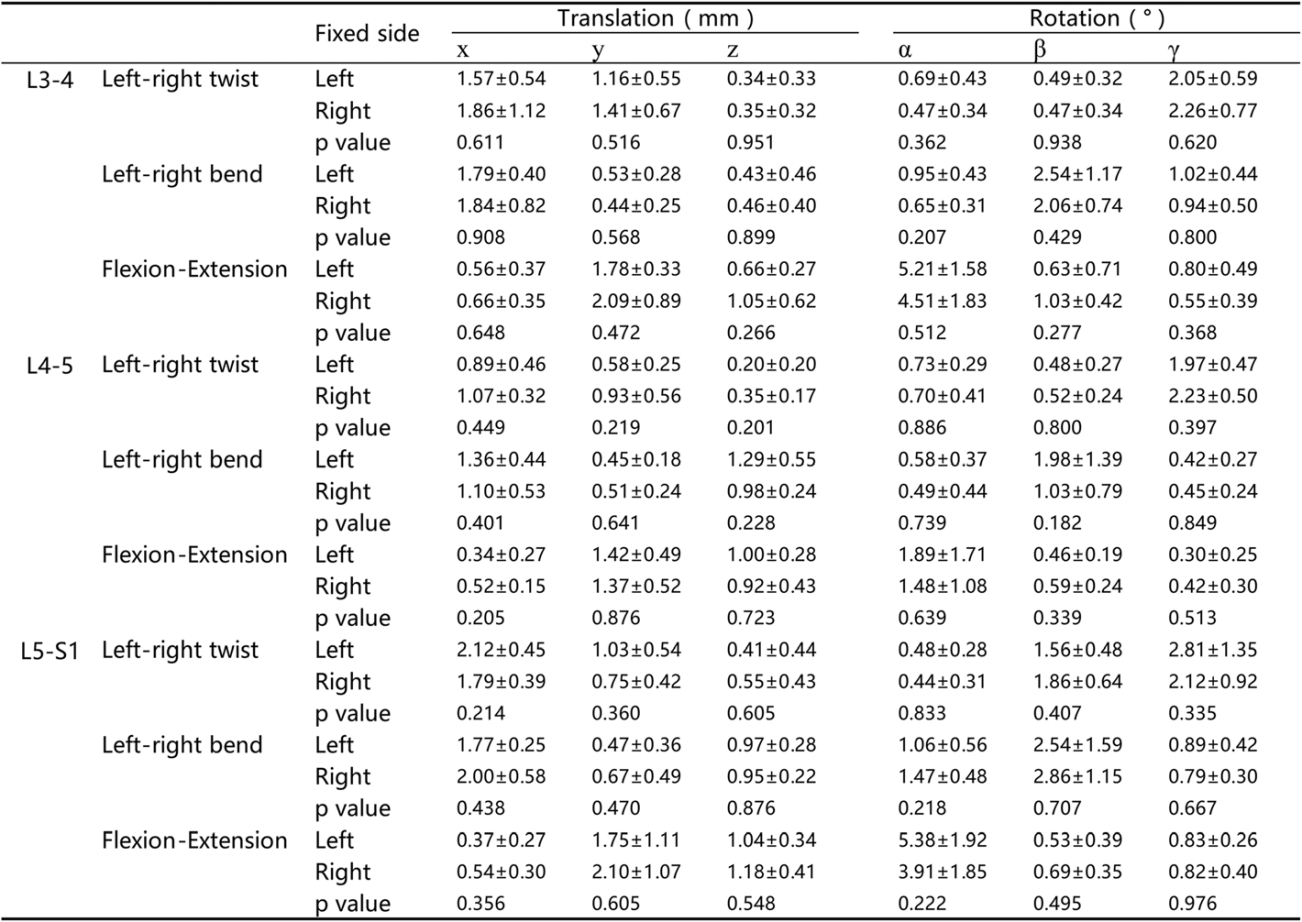
Invertebral 6DOF ROM of UPSF group with different fixed side during the different activities

Values are presented as mean ± SD

*x* left–right, *y* anterior–posterior, *z* proximal–distal translations, *α* flexion–extension, *β* left–right bending, *γ* left–right rotations

**Table 3:**
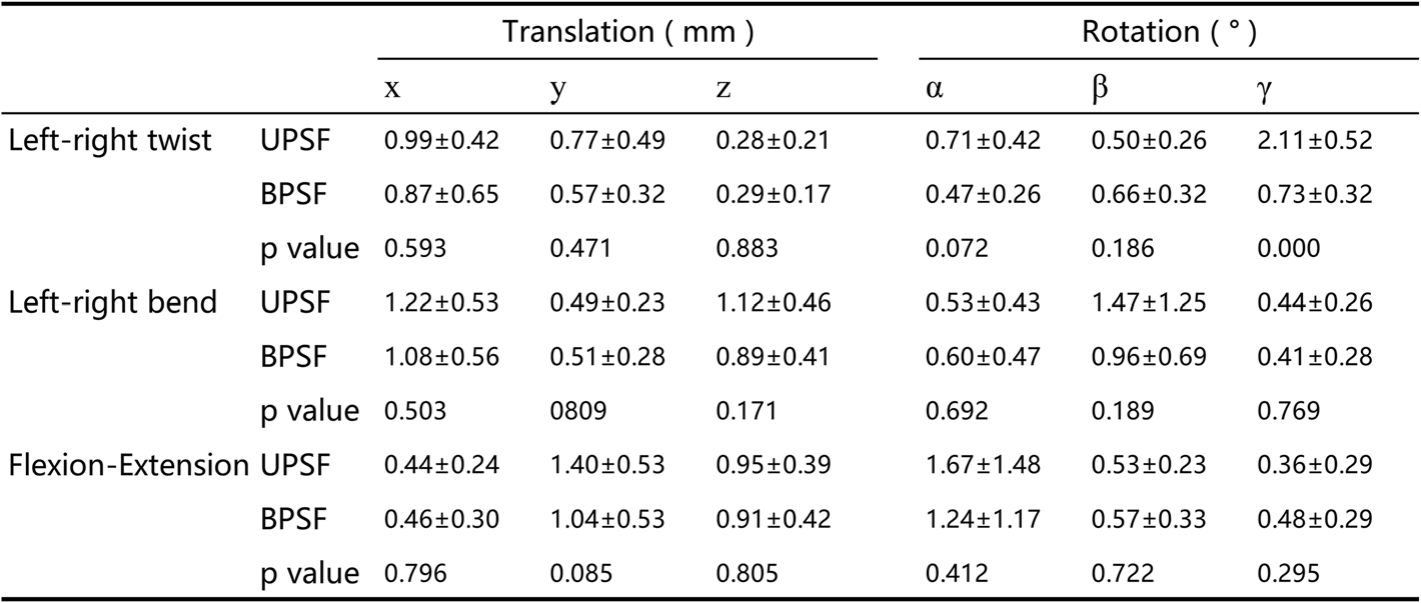
Invertebral 6DOF ROM during the different activities at L4–5 segment

Values are presented as mean ± SD

*x* left–right, *y* anterior–posterior, *z* proximal–distal translations, *α* flexion–extension, *β* left–right bending, *γ* left–right rotations

At the cranial adjacent segment L3–4, during left–right twisting, the primary ROM of the UPSF group (2.16 ± 0.73°) was significantly smaller (*p* = 0.003) than the BPSF group (3.17 ± 0.88°). During left–right bending, the UPSF group had a primary ROM of 2.28 ± 1.03°, that was significantly smaller than that (3.12 ± 1.04°) of the BPSF group (*p* = 0.041). During flexion–extension, there was no difference (*p* = 0.520) between the UPSF and BPSF groups (4.83 ± 1.82° and 5.29 ± 1.86°, respectively) (Fig. 3b). There were no statistical differences in coupled translations and rotations between the two groups (Table 4).

**Table 4:**
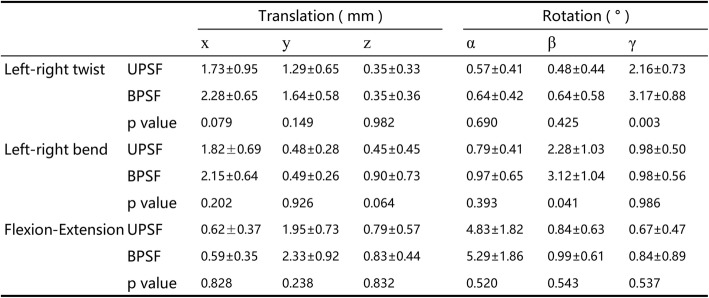
Invertebral 6DOF ROM during the different activities at L3–4 segment

Values are presented as mean ± SD

*x* left–right, *y* anterior–posterior, *z* proximal–distal translations, *α* flexion–extension, *β* left–right bending, *γ* left–right rotations

At the caudal adjacent segment L5–S1, there was no significant difference between the UPSF and BPSF groups in primary ROMs of left–right twisting (2.44 ± 1.24° vs 2.83 ± 1.23°, *p* = 0.413), left–right bending (2.71 ± 1.43° vs 3.16 ± 1.51°, *p* = 0.424) and flexion–extension (4.59 ± 2.10° vs 4.47 ± 2.31°, *p* = 0.890) (Fig. 3c). There were no differences between the two groups in all the coupled translations and rotations (Table 5).

**Table 5:**
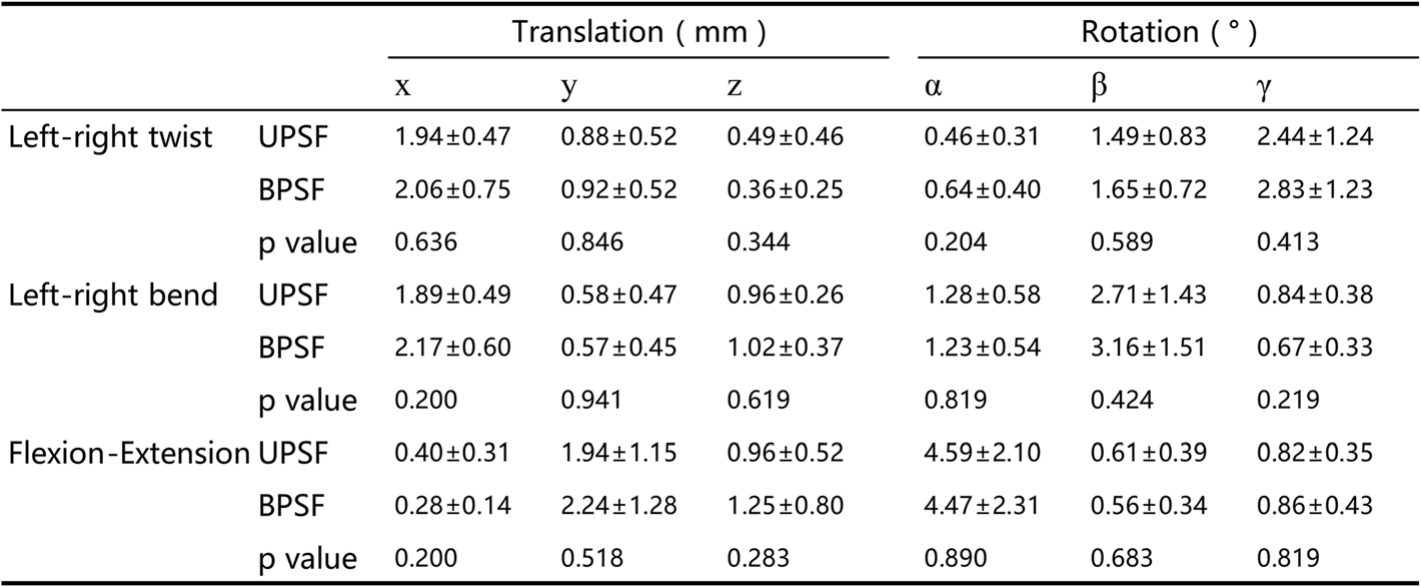
Invertebral 6DOF ROM during the different activities at L5–S1 segment

Values are presented as mean ± SD

*x* left–right, *y* anterior–posterior, *z* proximal–distal translations, *α* flexion–extension, *β* left–right bending, *γ* left–right rotations

## Discussion

This study investigated the ROMs of lumbar segments (L3–S1) of patients after fusion surgery of L4–5 using UPSF/TLIF or BPSF/TLIF. The data indicated that at the index L4–5 segment, the ROM in left–right twisting of the UPSF group was significantly higher than the BPSF group. In the cranial adjacent segment L3–4, the ROMs in left–right twisting and bending were significantly lower in the UPSF group at low flexion angles. No effect of the surgery was observed on the caudial adjacent segment L5–S1. The data proved our hypothesis that the UPSF is not as rigid as the BPSF in constraining the index segment, it could result in less effect on the adjacent levels compared to the BPSF technique.

According to previous studies, UPSF combined with TLIF can achieve similar clinical results for the treatment of lumbar DDD when compared with BPSF technique, but the UPSF has the advantages of smaller surgical trauma and lower hospital cost [7, 17]. In vitro biomechanical researches showed that UPSF did not provide sufficient stability for lumbar spine which may cause internal fixation failure and produce segmental intervertebral off-axis motion [8, 18]. For example, in the study of Slucky AV et al. [8], the surgical segmental ROM of UPSF group was significantly higher in the three directions of flexion–extension, left–right bending, and left–right twisting than the BPSF group. In the study of Harris BM et al. [19], they reported that compared with the BPSF group, the ROM in left–right twisting activity of UPSF group increased by 26% and 44% in left–right bending. In our study, we used the 2D-3D matching technique to compare the in vivo human spine 6DOF kinematics of UPSF and BPSF combined with TLIF for the treatment of L4–5 lumbar DDD. We measured the ROM of surgical segments (L4–5) and adjacent segments (L3–4 and L5–S1) simultaneously. At the L4–5 segment, we found that the ROM in left–right twisting of UPSF group was significantly higher than the BPSF group. There was no significant difference in left–right bending and flexion–extension between the two groups. Our results are partially consistent with previous in vitro biomechanical researches [8, 19]. Although the kinematics trends were similar among these studies, the ROMs of the two groups in this study were smaller than the previous in vitro biomechanical studies. This could be due to the different experimental setups between in vivo and in vitro studies. In vitro biomechanical experiments might not be accurate to replicate the activity of the spine under in vivo weight-bearing conditions, including the function of intervertebral ligaments and muscle contractions.

The purpose of a spinal internal fixation is to maintain the stability of the intervertebral graft. If the pedicle screw fixation is sufficiently rigid, it may result in implants loosing and intervertebral fusion failure. If the overall fixation is too rigid, it may accelerate the degeneration of adjacent intervertebral facet joints. Özkaya et al. [20, 21] performed biomechanics and kinematics study of UPSF on ovine vertebrae and found that unilateral dynamic and semi-rigid pedicle screw fixations can provide stability to the vertebrae and preserve both adjacent and fixed segments. Based on our previous study [7], although the short-term stability of the UPSF is not as rigid as the BPSF and may lead to prolonged fusion time, both surgical methods can achieve good fusion rates after more than 1 year follow-up. We therefore recommend longer time to wear the waist circumference after UPSF to promote postoperative recovery.

Previous studies demonstrated that the occurrence of adjacent segment degeneration after spinal internal fixation is inevitable, especially in cranial adjacent levels [22–24].

We found that the BPSF has a greater impact on the cranial adjacent level compared with the UPSF by measuring the disk height and the segmental lordosis on upright lateral digital X-rays [7]. At the present study, in the cranial adjacent level L3–4, the ROM of UPSF group was significantly lower than the BPSF group in the activities of left–right twisting and bending. However, there was no significant difference between the two groups in the flexion–extension, neither all three plane activities in the caudal adjacent level L5–S1. The stiffness of the BPSF in fusion segment is larger than the UPSF which may affect the mechanical loading of adjacent segments [9, 25]. This result implied that the UPSF could be more efficient to protect the contralateral facet joint during surgery and decrease the stresses at the cranial adjacent intervertebral facets and disc [26, 27]. This is one of the advantages of the UPSF that can reduce the effect of rigid spinal internal fixation on the adjacent vertebrae and the incidence of adjacent segment degeneration ultimately.

There are some limitations in the current study that should be noted. First, we only analyzed the low lumbar spines of L3–S1 after the surgery of L4–5 using UPSF or BPSF combined with TLIF. Future research should focus on the T12–S1 to observe the dynamic motion characteristics of whole lumbar spine. Second, we measured the lower lumbar motion in the short term after internal fixation in order to evaluate the stability of UPSF combined with TLIF compared with the BPSF techniques. Future studies should investigate the long-term effects of the UPSF and BPSF on adjacent segments. Third, the sample size was relatively small, although we detected the differences in ROMs cranial adjacent level (L3–4) and surgical segment (L4–5) in left–right twisting and bending. Finally, we had 6 left and 7 right UPSF patients. However, our post hoc analysis showed similar kinematics between the toe sub-groups.

## Conclusion

In summary, we applied the DFIS with 2D-3D matching technique to study the in vivo dynamic motion characteristics of low lumbar spine after UPSF and BPSF combined with TLIF for L4–5 lumbar DDD. The ROM in left–right twisting of UPSF group was significantly larger compared with BPSF group at the index level in the short term. The UPSF has less impact on the cranial adjacent level (L3–4) in left–right twisting and bending activities compared to the BPSF. The data implied that the UPSE and BPSF combined with TLIF would result in different biomechanics in the index and cranial adjacent segment biomechanics. Long-term follow-up studies are necessary to compare the clinical outcomes of the two surgeries.

## Additional file

Additional file 1: Comparison of the VAS, ODI, and JOA score of the UPSF group with different fixed side. (TIF 1051 kb)

## Data Availability

All data generated or analyzed during this study are included in the manuscript.

## Abbreviations

6DOF: 6 degrees of freedom
BPSF: Bilateral pedicle screw fixation
CT: Computed tomography
DDD: Degenerative disc diseases
DFIS: The dual fluoroscopic imaging system
ROMs: Ranges of motion
TLIF: Transforaminal lumbar interbody fusion
UPSF: Unilateral pedicle screw fixation
