# Supplementary figures and images for: In vivo dynamic motion characteristics of the lower lumbar spine: L4–5 lumbar degenerative disc diseases undergoing unilateral or bilateral pedicle screw fixation combined with TLIF
Source: J Orthop Surg Res. 2019 Jun 7;14:171. doi: 10.1186/s13018-019-1198-6 (PMC6555927; doi:10.1186/s13018-019-1198-6)

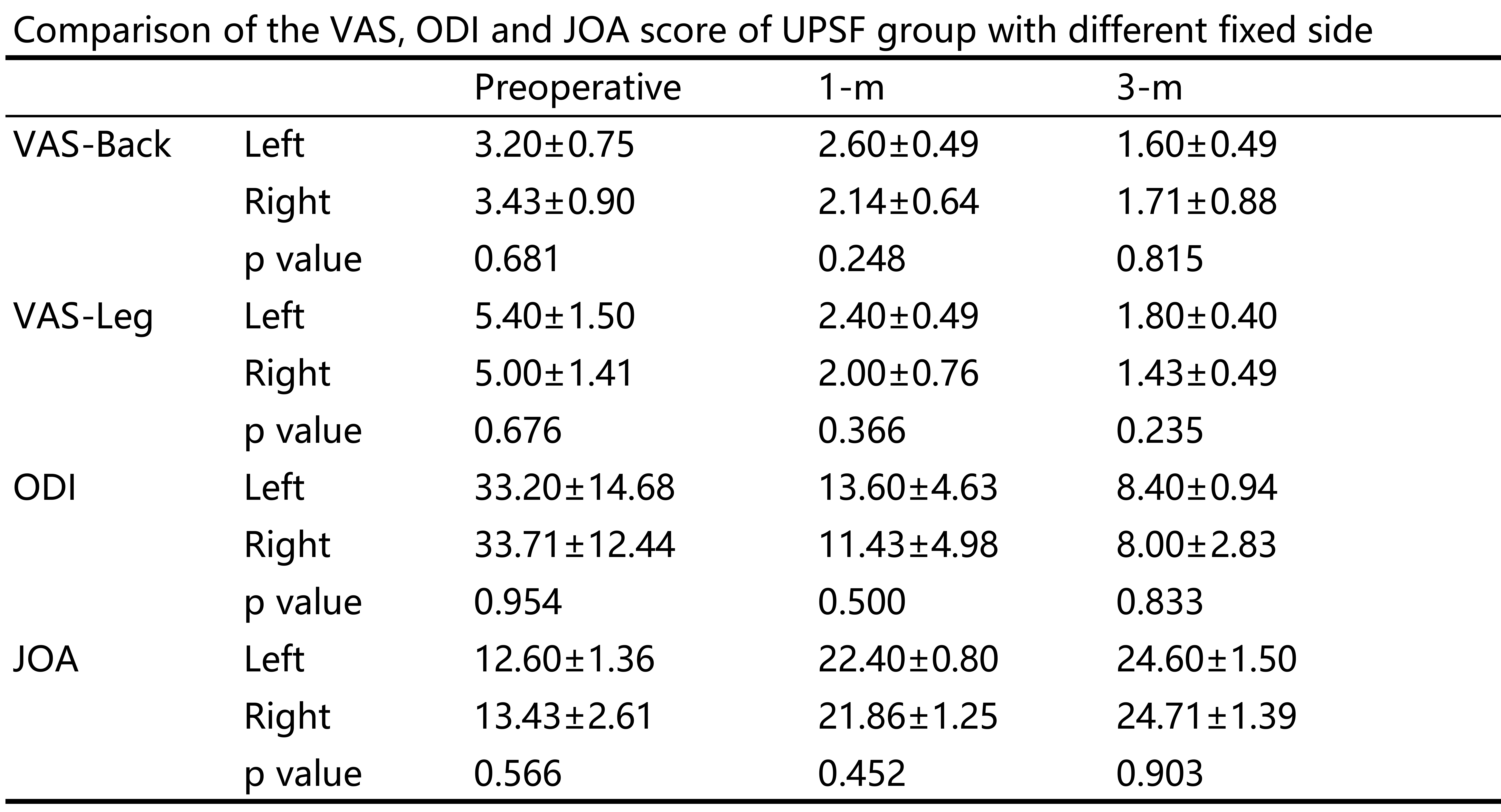

Supplement: Supplementary file 1 — Comparison of the VAS, ODI, and JOA score of the UPSF group with different fixed side. (TIF 1051 kb) [file 13018_2019_1198_MOESM1_ESM.tif]
